# Assessing the Potential Deployment of Biosensors for Point-of-Care Diagnostics in Developing Countries: Technological, Economic and Regulatory Aspects

**DOI:** 10.3390/bios8040119

**Published:** 2018-11-29

**Authors:** Daniel Migliozzi, Thomas Guibentif

**Affiliations:** 1 Laboratory of Microsystems, Ecole Polytechnique Fédérale de Lausanne, 1015 Lausanne, Switzerland; 2 Energy Center, Ecole Polytechnique Fédérale de Lausanne, 1015 Lausanne, Switzerland; thomas.guibentif@alumni.epfl.ch

**Keywords:** point-of-care diagnostics, low-resource settings, patterned-paper technology, technological breakthrough, medical biosensors, cost-effectiveness

## Abstract

Infectious diseases and antimicrobial resistance are major burdens in developing countries, where very specific conditions impede the deployment of established medical infrastructures. Since biosensing devices are nowadays very common in developed countries, particularly in the field of diagnostics, they are at a stage of maturity at which other potential outcomes can be explored, especially on their possibilities for multiplexing and automation to reduce the time-to-results. However, the translation is far from being trivial. In order to understand the factors and barriers that can facilitate or hinder the application of biosensors in resource-limited settings, we analyze the context from several angles. First, the technology of the devices themselves has to be rethought to take into account the specific needs and the available means of these countries. For this, we describe the partition of a biosensor into its functional shells, which define the information flow from the analyte to the end-user, and by following this partition we assess the strengths and weaknesses of biosensing devices in view of their specific technological development and challenging deployment in low-resource environments. Then, we discuss the problem of cost reduction by pointing out transversal factors, such as throughput and cost of mistreatment, that need to be re-considered when analyzing the cost-effectiveness of biosensing devices. Beyond the technical landscape, the compliance with regulations is also a major aspect that is described with its link to the validation of the devices and to the acceptance from the local medical personnel. Finally, to learn from a successful case, we analyze a breakthrough inexpensive biosensor that is showing high potential with respect to many of the described aspects. We conclude by mentioning both some transversal benefits of deploying biosensors in developing countries, and the key factors that can drive such applications.

## 1. Introduction

On the front line, controlling the spread and impact of infectious diseases is a challenging health issue in remote low-resource environments [1]. Moreover, surging of antibiotic resistance [2,3] and rising costs of effective drugs [4] are associated issues that have led to increased attention towards alternative methods for diagnostics. In the emblematic case of malaria, a well-known burden in remote regions, microscopy and lateral-flow assays (LFA) are the only available diagnostic tests in these regions [5,6,7]. Nevertheless, none of them are truly suitable: microscopy is quantitative but expensive due to the highly sophisticated laboratory tools required [8,9], whereas LFA is cheaper but only qualitative. On the treatment side, the cost of artemisinin therapy is increasing [4] and drug-resistance is surging in *plasmodium* species due to unadapted treatments [10]. Another challenge of health systems in low-resource environments is blood transfusions: hepatitis and HIV viruses in blood samples cause infection transmission and wastage of samples [11,12]. 

The biosensing sector has been actively explored in the past decades, both for new applications and for new markets, from genome sequencing [13], to drug screening [14], from Alzheimer diagnostics [15] to nutrient monitoring in blood [16]. As a matter of fact, the large majority of the businesses target usage in developed countries. In contrast, an orphan context is that of developing countries. Obviously, the latter have needs that are much different from the former, but many of these needs are still unfulfilled, creating a potential for biosensor applications that remain, thus far, unexplored. Especially, the lack of well-equipped medical infrastructures along with the frequent necessity to run decentralized tests in remote sites to decrease the time-to-results (e.g., to diagnose infections in isolated villages or to detect pathogens in water basins) are crucial issues that can benefit from technologies that enable such point-of-care testing.

However, to understand whether the modern biosensing devices may be suited for the healthcare sector in developing countries, one has to take into account technical features, but also mainly the lack of qualified personnel and infrastructures in low-resource settings, along with costs, financing and regulatory aspects. In the following chapters, we describe the conceptual structure of a biosensor and we assess the main technical, regulatory and economic factors which influence their potential use to meet sanitary needs in developing countries. Finally, we study the example of the patterned paper technology [17] as a game-changing biosensing solution.

## 2. Part I. A Biosensor “Shell by Shell”

A commonly used definition for a biosensor is that of a device that detects, and often quantifies, targets of several nature (e.g., pathogens, disease markers, physiological parameters) using one or more components of biological or biomimic origin. Research in this field is extremely active both in academic and private laboratories, resulting in a variety of devices and applications coming to light almost daily. Here, we aim at classifying a biosensor in a way that is independent of its core technology and its target application. For this, we decompose the device into four functional shells, which often translate into physically distinct components, each having a different role in transferring the information from the target up to the user:

1. a *sensitive element* is used to create an interaction between the target and the device. This interaction should involve the target as selectively as possible compared to other components of the sample, in order for the information to be specific to the target. For this part, biological molecules such as DNA strands, aptamers, and antibodies are a particularly adapted option, given their high specificity to the most widely demanded analytes;
2. a *transducer* is used to convert the interaction effect into a readable parameter. Fluorescent molecules and functionalized surfaces (where usually organic molecules are bound to solid materials) are examples of such components, used in a large variety of biosensors;
3. a *detection system* is used to measure the signal coming from the interaction between the device and the target. For instance, elements such as filters, lens, photodiodes and CCD cameras are widely used when the readout is optical, whereas silicon or metal components are common when the readout is electrical;
4. a *transporter* includes the whole structure in which the other elements are contained. This part may also integrate steps for sample pre-processing (centrifugation, extraction, sorting, etc.) and data analysis which does not involve the biosensing step *stricto sensu*. The transporter can be very bulky like a fully integrated genome sequencer [18], down to simple assemblies contained in cm-sized tools [19].

**Figure 1:**
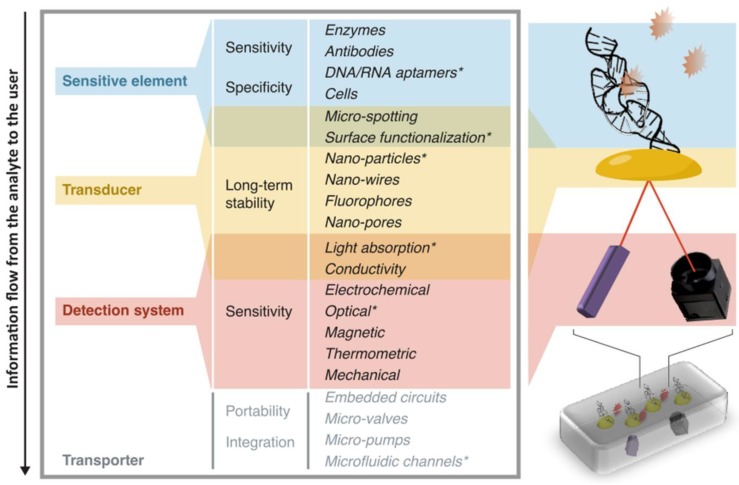
Functional partitioning of a biosensor. Schematic view of the parts that compose a biosensor, conceptually and physically, along the information flow from the analyte to the user. Each functional partition is indicated with a specific color. For each partition, we indicate qualities that are crucial for implementation in low-resource settings, and a list of components/properties specific to that partition. Elements marked with * are the ones illustrated in the picture on the right, which compose an example of a portable biosensor described in [19].

This analytical partitioning is very useful to identify and understand the assets and the issues related to the different parts of the information flow from the target to the user. Figure 1 shows a schematic view of this classification, with some keywords related to features involving each of the device partitions. To exemplify the structure above, we describe here two biosensors that use different technologies and readouts:

- In the case of a portable SPR sensor as proposed by Guiducci and co-workers [19], the goal is to quantify the concentration of tobramycin (an antibiotics) filtered from undiluted blood serum. The sensitive element is a DNA-based aptamer. Many copies of this DNA strand cover isolated gold nano-islands, which change their refractive index upon binding of the complementary strands, and thus act as transducers. The detection system can then measure this change by using a light source for excitation and a photo-detector. The transporter is then a glass slide for the microfluidic handling of the sample and a plastic box in which all these elements are assembled. A schematic view of the functional parts of this portable biosensor is illustrated in Figure 1.
- In the case of the PacBio RS II by Pacific Biosciences [18], the goal is to sequence long DNA strands, for mutation detection or gene expression quantification. The sensitive element is composed of polymerase proteins trapped into nanowells. The transducer is a set of dyes with which single nucleotides are tagged. The detection system consists of an evanescent excitation system able to measure the fluorescence of single nucleotides in the nanowell, which changes over time during their interaction with the polymerase. The transporter is a large structure that integrates all the opto-electronics for the sensing and the hardware for the analysis.

## 3. Part II. Technological Challenges in Low-Resource Settings

To be applicable in and beneficial for developing countries, biosensors have to fulfil many major operational requirements, which are specific to low-resource settings. First, because of poor external quality control and unreliable procurement systems the proposed biosensors should require as few external reagents and as little external instrumentation as possible. Second, due to the lack of essential laboratory equipment to perform control tests, the results need to be highly reproducible. Ideally, the devices should operate fully autonomously (as a “black-box”) in a robust way for a long time. Upstream of the point of use, conservation of both the device and the disposables has to be guaranteed for the long-term, even in harsh conditions, because of the scarce refrigeration and power supply capacities in remote locations. Downstream, because of the poor waste-management facilities present in low-resource settings, all the disposables of the device should have a very low environmental impact.

Many of these challenges have already been considered by current biomedical R&Ds, which try to conceive highly integrated and portable biosensors that are very user-friendly and have very little reagent consumption. This is made possible by several factors: the miniaturization of the elements of the detection system [20,21], the optimized micro-structuring of the transducer (e.g., nano-wires [22], nano-pores [23], nano-particles [24]), the microspotting of the sensitive element [25], and the integration within the transporter (e.g., with low-volume-consumption microfluidics [26]). Furthermore, the recent widespread utilization of smartphones as imagers [27] can enable field analysis by wireless communication between remote sites and centralized labs, and give the opportunity to perform some field tests (e.g., detection of pathogens in the water), which can definitely prove interesting for developing countries. Another valuable technical feature of biosensors is their potential flexibility in terms of analytical capability. For instance, when the purpose is to detect an infectious pathogen in the blood, a biosensor capable of testing for several pathogens (at the same time or one of a few options) would indisputably be an asset. Nowadays, this versatility is made possible by many multiplexing technologies that allow hundreds of biomarkers to be quantified at once [28]. 

Yet, there is a drawback to these multiple possibilities: the trend for current R&Ds to work towards disparate and complex applications in order to demonstrate the full range of usages slows down the development, defocuses from the industrial scalability and delays the deployment of the product. If developing countries are to benefit from biosensors, there is an equilibrium to find between the quest for the best technical solution and the one that is best suited for poor environments with urgent medical priorities: addressing concrete and immediate needs would definitely be key for successful deployment. 

However, even for technologies reaching a satisfactory maturation state regarding technical criteria, it appears that limiting steps in the biosensing workflow are often upstream and downstream of the biosensor use itself. On the upstream side, the challenges of sample collection and storage are often underestimated when proposing solutions for developing countries. In fact, in order for the sample collection and preparation to be properly performed, the action of a skilled operator and/or the use of specific equipment are often necessary (for blood collection, for instance). Moreover, when not directly prepared and analyzed, the samples are almost always required to be stored under highly controlled conditions. On the downstream side, using biosensors does not obviate the guidance of a doctor who interprets the results (namely relating them with other symptoms) and gives prescriptions or asks for further analysis. Some diagnosis cannot be done with biosensors alone anyway and false positives/negatives are never to be excluded.

Therefore, a network of competent personnel adapted to the level of complexity of use and handling not only of the biosensor product but also of the samples and the choice of the subsequent treatments should be set up in parallel to their distribution. If this is not taken into account early enough in the implementation process, there is a risk for remote regions to be flooded with “gadget-biosensors" before the necessary competencies to handle that resource have been created. This could lead, among others, to misdiagnosis, wasted devices, and pollution. Even if this is not direct responsibility of researchers and involved companies, these factors should be considered as early as possible to better target relevant and practicable applications. Conversely, there is an important role to be played by governments and supervising organizations both as financing providers and regulators, to put in place all the human and material resources.

## 4. Part III. Tackling Financing and Regulations

Despite their attractive potential, biosensing technologies already struggle to exit laboratories and enter real-world applications even in developed countries. One example that has been observed with much attention in the past decade is the genome sequencing test [29], the cost of which decreased from 100 M$/subject to 1 k$/subject in less than 15 years [30]. This shows how the private sector has the capability to decrease the costs of techniques in order to address specific applications. In many cases, however, even when the core technology has been brought down to its minimal cost, it usually results in products that would be unaffordable in low-resource settings. This occurs because, since in developed countries biosensors are usually designed to meet business profitability, the main approach to cost reduction is the continuous amelioration of high-tech processes. Conversely, investment in inexpensive breakthrough technologies is rarely chosen in wealthy settings. However, a bottom-up approach based on inexpensive technological solutions could prioritize affordability, and therefore be more suited to target applications for developing countries. Such an approach can be enabled by the use of inexpensive materials [31] and open-source components [32,33,34]. Free designs for hardware such as microfluidic modules [35] and microcontrollers [36] may help in creating a cheap transporter, for instance. Free schemes and procedures are also available for high-resolution optical detection [37] and image-processing [38], which could be very useful when conceiving the detection system. This new development paradigm based on building upon open-source bricks has the potential to greatly reduce the cost of usually highly customized solutions. However, most stakeholders have financial interests in protecting their know-how and are not often prone to such a community-driven approach.

However, the cost of the tests has to be regarded along with other important factors: disease prevalence, cost of treatment, cost of mistreatment, and throughput. Indeed, cost-effectiveness for diagnostic tests considers that improvement in accuracy and in time-to-result of the current tests can reduce the unneeded presumptive treatments and better harness the necessary ones, which would be an increased net gain for resource-limited settings. Therefore, an acceptable price depends on the benefits: cheaper is not always better, and one should also take into account the policy makers’ valuation of a disability-adjusted life-year (DALY) in those countries to set the approximate value-cost of a diagnostic device. 

Besides the cost issue, there is also a double challenge on financing: on one hand, biosensors development receives much less financial support than do drug and vaccine development; on the other hand, the healthcare system in developing countries does not often support the clinics with reimbursements for tests and treatments, which most of the patients cannot pay by themselves. Significant funding is coming from United States government (mainly for pathogen diagnostics driven by biodefense concerns [39]), but dedicated resources from national programs, non-profit organizations, international agencies and donations would likely not cover the needs for both the development of biosensor devices and their retail. An additional financing source could be that the private sector itself subvents the early adoption of biosensors in test regions. This may demonstrate the effectiveness of the devices and pave the way for further investments. 

Lastly, beyond this financing barrier to the diffusion of biosensors in developing countries, there are high regulatory barriers due to the strict international healthcare standards. Companies are under continuous pressure to obtain the certifications required for the adoption of their products, in a highly competitive environment. For example, licenses are issued on genes and molecules, which makes it increasingly expensive for new companies to build upon existing technologies to create diagnostic tools using those genes or molecules. Moreover, while laboratories regularly present promising new technologies, it is technically difficult to reach good reproducibility of results in the final prototype, which may prevent from fulfilling the standards needed to comply with the regulation. Furthermore, even when all the resources and efforts are put in place for this purpose, other obstacles may occur independently from the market and the local decision makers, such as delays, inconsistency and unpredictability of medical agencies [40]. Furthermore, as developing countries do not usually have standards to evaluate diagnostic tests, it is difficult for the state and the medical infrastructure to select among possible alternative tests for purchase. Also, a habilitated person has to take the legal responsibility of interventions subsequent to a biosensor test (e.g., treatment, supplementary tests). Once again, the need for implementing a network of qualified personnel and adapted frameworks and legislations is crucial to deploy biosensors in low-resource settings, even when the technical and economic requirements are met. 

## 5. Part IV. A Technological Breakthrough as a Promising Case

All the previous factors make the deployment of biosensors in developing countries very hard. Thus, companies have low or even no interest in spending time and resources in designing affordable products for poor settings, which still remain unappealing high-risk markets for the applications that we already meet in developed countries. Nevertheless, an example of solution that has a potential for success in terms of economic sustainability is the patterned paper technology (PPT) developed by Diagnostics For All, a not-for-profit foundation whose goal is to provide ultra-low-cost biosensors for the healthcare sector in developing countries [41]. Instead of trying to decrease the cost of current technologies, their key strategy is to create breakthrough inexpensive biosensors based on simple paper [17]. This technology is enabled by three-dimensional wax patterning [42,43], and was shown to be suitable for ELISA assays [44]. The applications they currently focus on are infectious disease diagnostics, liver toxicity assessment, bovine heat detection, child nutrition, and vaccination.

By using the partition we proposed in Part I, we present the case of their paper-based sensor for alanine transaminase [45] (ALT), of which the blood level is indicative of the liver function:

5. the *sensitive element* is L-alanine, which is converted to pyruvate by ALT;
6. the *transducer* is a red dye formed as an end-product of a series of enzymatic reactions starting by the pyruvate;
7. the *transporter* is a piece of paper treated with wax to integrate channels for sample handling and chambers for reaction detection;
8. the *detection system* is merely the eye of the operator, who analyses the result with the help of a simple color-scale, and positive/negative controls integrated on the paper chip.

**Figure 2:**
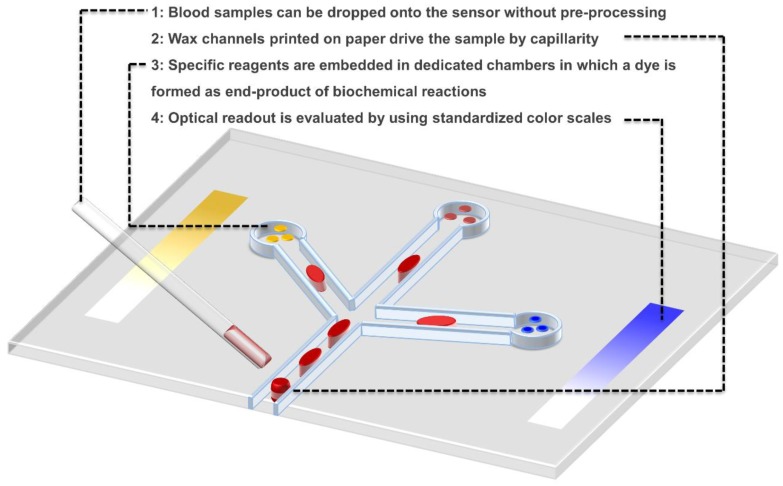
Patterned Paper Technology. Schematic of the technology described in Part IV to sense alanine transaminase in blood.

This very simple device (Figure 2) has already been characterized in terms of accuracy and specificity [46], and tested on clinical cases [47]. It obtained the Establishment Registration from the Food and Drug Administration and is currently under submission for regulatory approval for Point-of-Care use [48]. 

In Table 1 we report a Strengths-Weaknesses-Opportunities-Threats (SWOT) analysis of this technology in view of the deployment in the healthcare sector in developing countries. The main strengths of this paper device are the very low cost (in the order of pennies) and the ease of use by a non-specialized operator. Also, the simplicity of the readout allows the doctor to make straightforward interpretation to address subsequent actions. There are uncountable applications, thus opportunities, for such sensors in developing countries, related to the many sanitary needs that remain unmet. The major weaknesses are, on the one hand, the fact that highly quantitative information is unlikely to be accessible with this technique (at least at this stage) and, on the other hand, the extreme fragility of the device itself. Potential threats may come from the unavailability of direct post-diagnostic treatments in developing countries, which makes many technically feasible tests useless, and that much of private and public investments go to drug and vaccine development rather than to diagnostics devices. 

## 6. Perspectives

Beyond a faster and more reliable way to diagnose, another positive by-product of deploying biosensing devices conceived for low-resource environments includes the creation of better epidemiological data, which are important for several reasons:

- to model infectious disease spreading,
- to understand drug resistance surging,
- to efficiently introduce vaccinations,
- and to define strategies for the healthcare system (for medical intervention in specific areas, for sanitary reimbursements, etc.).

Moreover, the sanitary needs in developing countries are not limited to human diagnostic purposes. Indeed, food safety may also be considered as a potentially interesting sector to which biosensors can contribute: quality control of seed and water may have a huge impact on people’s health in poor settings. 

It seems likely that the deployment of biosensors can widen in developing countries following some crucial factors:

- the research in breakthrough inexpensive technologies;
- the sharing of open-source technical solutions;
- the improvement of low-cost fabrication processes and scalability;
- the rising engagement of regulatory agencies in the clinical validation of the promising devices;
- and the commitment of local medical staff to modify their practices towards innovative diagnostic methods.

## Figures and Tables

**Table 1:** List of the main strengths, weaknesses, opportunities and threats for the patterned paper technology described in Part IV related to its deployment in developing countries.

***Strengths—Internal Positive Factors*** Ultra-low-cost (less than 1$) Power-supply-free Safe disposable Portable No need of specialized operator Equipment-free Multiplexing capability	***Weaknesses—Internal Negative Factors*** Not quantitative Fragile Single-use Very basic sample processing only
***Opportunities—External Positive Factors*** Many addressable needs (e.g., infectious diseases diagnostics, vaccination optimization, nutritional monitoring). Reimbursement from the state may motivate hospitals/patients to make use of them.	***Threats—External Negative Factors*** Regulatory agencies may delay/oppose clinical validation Much fewer investments than in drug discovery and vaccine development Slow adoption by physicians and mistrust of results Low demand because of low revenue of the patients
